# A Revisiting Method Using a Covariance Traveling Salesman Problem Algorithm for Landmark-Based Simultaneous Localization and Mapping

**DOI:** 10.3390/s19224910

**Published:** 2019-11-10

**Authors:** Hyejeong Ryu

**Affiliations:** Department of Mechatronics Engineering, Kangwon National University, Chuncheon KR24341, Korea; hjryu@kangwon.ac.kr; Tel.: +82-33-250-6376

**Keywords:** mobile robot, simultaneous localization and mapping, traveling salesman problem, path planning, loop-closing, exploration

## Abstract

This paper presents an efficient revisiting algorithm for landmark-based simultaneous localization and mapping (SLAM). To reduce SLAM uncertainty in terms of a robot’s pose and landmark positions, the method autonomously evaluates valuable landmarks for the data associations in the SLAM algorithm and selects positions to revisit by considering both landmark visibility and sensor measurement uncertainty. The optimal path among the selected positions is obtained by applying the traveling salesman problem (TSP) algorithm. To plan a path that reduces overall uncertainty, the cost matrix associated with the change in covariance between all selected positions of all pairs is applied for the TSP algorithm. From simulations, it is verified that the proposed method efficiently reduces and maintains SLAM uncertainty at the low level compared to the backtracking method.

## 1. Introduction

Environmental mapping is essential when mobile robots are used to perform higher-level tasks. Many researchers have explored how to present the environment and localize a robot using sensor data. Simultaneous localization and mapping (SLAM) is used to simultaneously estimate the positions of a robot and landmarks from noisy sensor data acquired as the robot moves [1,2]. Many SLAM algorithms focus on the accuracy of estimated states; various filters and frameworks have been applied to bound the associated uncertainties.

Feature- and landmark-based SLAM approaches usually employ extended Kalman filters (EKFs) to estimate the status of the robot and landmarks and employ a single Gaussian for each state. In such approaches, line or point landmarks are extracted from data derived by laser range finders, sonar sensors, and visual landmarks in images [3,4]. The performance of SLAM algorithms depends on process and measurement noise covariance matrices. To obtain accurate measurement noise covariance matrices without the trial and error methods of EKF-SLAM algorithms, an adaptive neuro-fuzzy inference system was proposed [5]. To reduce the computational complexity of EKF-SLAM, which is affected by the number of landmarks, local submaps are constructed and they are integrated into a global map [6]. Microphone observations can be applied to landmark-based EKF-SLAM algorithms [7]. The time delay estimation between sound sources and microphones by generalized cross-correlation was used to establish the observation model.

Rao–Blackwellized particle filters (RBPFs) are appropriate for landmark-based maps [8], and also efficiently optimize gridmaps and estimate robot poses [9,10]. Radio frequency identification (RFID) phase measurements were employed to estimate the robot pose and passive tag coordinates by using the landmark-based RBPF SLAM approach [11]. Whereas most previous gridmap-based RBPF SLAM algorithms have modeled each cell as one Bernoulli distribution [12,13], the occupancy probability of each cell is modeled by using a beta-distribution in β-SLAM to distinguish between different causes of uncertainty [14]. It also presents an uncertainty measure to quantify the resulting gridmap by the Shannon entropy for path planning.

Recently, graph-SLAM algorithms have found many applications in large-scale environments. Graph-SLAM [15] optimizes the robot pose graph using constraints that connect the pose nodes via sensor observations. The sensor observations of the graph-SLAM algorithms can be the scan-matching results between laser point clouds [16,17] and the feature-matching/tracking results between keyframe images [18,19]. Keyframe-based graph-SLAM was applied not only to a single robot, but also to multi-agent systems [20]. The graph-SLAM algorithm performed by multiple robots can overcome the inefficiency caused by the growing graph size in large environments. To increase the performance of the graph-SLAM algorithm for low-cost Light Detection And Ranging (LiDAR) sensors and vision sensors, laser scan-matching results and visual bag of words (BoW) were combined [21].

When using SLAM algorithms, loop-closing (i.e., revisiting a previously mapped/detected area) is essential to bound global errors and to improve global consistency. Revisiting minimizes SLAM uncertainty in terms of both the robot pose and landmark positions [22,23]. In general, when a robot returns to the initial position (the startpoint of the SLAM algorithm), a loop trajectory is naturally completed and the SLAM state is optimized by applying the loop-closing constraint [24,25]. This is because, when the SLAM algorithm commenced, the robot pose uncertainty was very low and landmarks were accurately registered. Robot and landmark uncertainties become large when the robot has traveled a long distance, necessitating return of the robot to a better-known area to re-observe accurately estimated landmarks; this increases the likelihood that data associations will be correct. Most loop-closing strategies have focused on detection of loop-closing landmarks and loop optimization [21,26,27]. Several approaches simplify loop-detection issues using special landmarks [11,28]. Appropriate loop-closing positions and planning of loop-closing paths have received less attention.

When a SLAM algorithm must reduce state uncertainties via loop-closing, the robot can easily return to the initial position if it is not remote from that position. However, if the robot is distant from the initial, accurately mapped area, the robot has to backtrack along the previous trajectory or plan a revisiting path that takes the robot in a direction in which uncertainty decreases. It is difficult to determine the extent to which uncertainty is reduced during backtracking, because the same situation inducing growing uncertainty is repeated along the backtracking path.

In this paper, the revisiting method using a solution of the traveling salesman problem (TSP) to devise a landmark-based EKF SLAM is proposed. Given the uncertainties in the positions of SLAM-registered landmarks when revisiting is to commence, we construct a visible landmark uncertainty map and select several revisiting positions to ensure that the obtained measurements adequately reduce uncertainty. The traveling order among the selected positions is chosen via TSP optimization using the predicted covariance changes. We show that robot and landmark uncertainties are reduced more efficiently when the robot travels along such a path (compared to backtracking).

This paper is organized as follows. In Section 2, we propose the visible landmark uncertainty grid map that is used to identify landmarks that must be re-observed to reduce overall uncertainty and to select appropriate revisiting positions. In Section 3, we present the covariance TSP algorithm to derive the revisiting path that includes all selected positions. Section 4 shows simulation results of both backtracking and our method. Section 5 is the conclusion.

## 2. Visible Landmark Uncertainty Portrayed on a Gridmap

To choose positions at which a robot can make loop-closing observations of landmarks registered by the SLAM algorithm, we construct a visible landmark uncertainty (VLU) map that shows the uncertainties of visible or observable landmarks at each grid position.

### 2.1. The VLU Map

The VLU map is a gridmap; the value of each grid is the extent of uncertainty associated with landmark detection at each grid location. We use the covariance matrix of the EKF-SLAM to represent the uncertainty of the SLAM state. This includes correlations between interrelated landmarks. Every observation of a landmark affects the estimation of every other landmark [1]. Therefore, it is optimal to proceed to a position at which the maximum number of possibly known or registered landmarks is visible to the SLAM algorithm, and where new landmarks are also detectable as exploration proceeds. To build the VLU map, we assume that the robot is equipped with a range sensor with a 360${}^{\circ }$ sweep but a defined maximum range. We first calculate the number of visible landmarks in each grid cell, $G_{i,j}$:

(1) $$G_{i,j}=\sum_{n=1}^{N}v_{n}$$

(2) $$\begin{array}{c} v_{n}=\left\{\begin{array}{cc} 1, & \mathrm{if}\sqrt{{\left\{g_{i,j}\left(x\right)-m_{n}\left(x\right)\right\}}^{2}+{\left\{g_{i,j}\left(y\right)-m_{n}\left(y\right)\right\}}^{2}}<r_{\max}\\ 0, & \mathrm{otherwise} \end{array}\right. \end{array}$$

where *N* is the total number of landmarks registered to the SLAM state, $g_{i,j}\left(x\right)$ and $g_{i,j}\left(y\right)$ are the metric coordinates *x* and *y* of the grid index $\left(i,j\right)$, $m_{n}\left(x\right)$ and $m_{n}\left(y\right)$ are the metric coordinates of the *n*-th landmark $m_{n}$, and $r_{\max}$ is the maximum range of the sensor. After calculating the number of visible landmarks, the VLU of each grid cell is derived as follows:

(3) $$\begin{array}{c} VLU_{i,j}=\left(N-G_{i,j}\right)tr_{\max}+\sum_{k=1}^{G_{i,j}}trace\left(P_{k}\right) \end{array}$$

(4) $$\begin{array}{c} tr_{max}=\max\left\{trace\left(P_{k}\right):k=1,\cdots ,N\right\} \end{array}$$

where *N* is the total number of landmarks registered to the SLAM state, $G_{i,j}$ is the number of visible landmarks at grid index $\left(i,j\right)$, $P_{k}$ is the covariance matrix of the visible landmark $m_{k}$, and $trace\left(P_{k}\right)$ is the trace of the $P_{k}$ matrix. $P_{k}$ is obtained from the SLAM covariance matrix; $P_{k}$ is a sub-block of the entire covariance matrix. $tr_{\max}$ is the maximum trace (derived by reference to the most uncertain landmark among all registered landmarks).

The Shannon entropy (the Shannon information measure) was used in [29] to obtain a scalar reflecting the diversity of a probability distribution represented by a covariance matrix. The entropy *H* of a Gaussian SLAM state is obtained by calculating the determinant of the covariance [30]. Building on this concept, the authors of [31] proposed the use of an *a-optimal* measure of information gain to increase map quality during exploration. The *a-optimal* measure is calculated using the trace of the covariance matrix, thus the product of the eigenvalues. When moving in a direction that optimizes the *a-optimal* measure, a more accurate map is obtained (the mean squared errors of the landmarks are minimized).

In Equation (3), we apply this trace of the state covariance matrix to the visible landmarks. The first term ($N-G_{i,j}$) reflects the number of invisible landmarks, and the second term reflects the uncertainties of the visible landmarks. The maximum trace for each invisible landmark is added, and each visible landmark assumes its corresponding trace.

The higher the grid cell VLU value, the greater the SLAM uncertainty; fewer landmarks with large covariances are visible. The lower the grid cell VLU, the better the robot position; covariance is reduced via correct association of data with visible landmarks.

**Figure 1:**
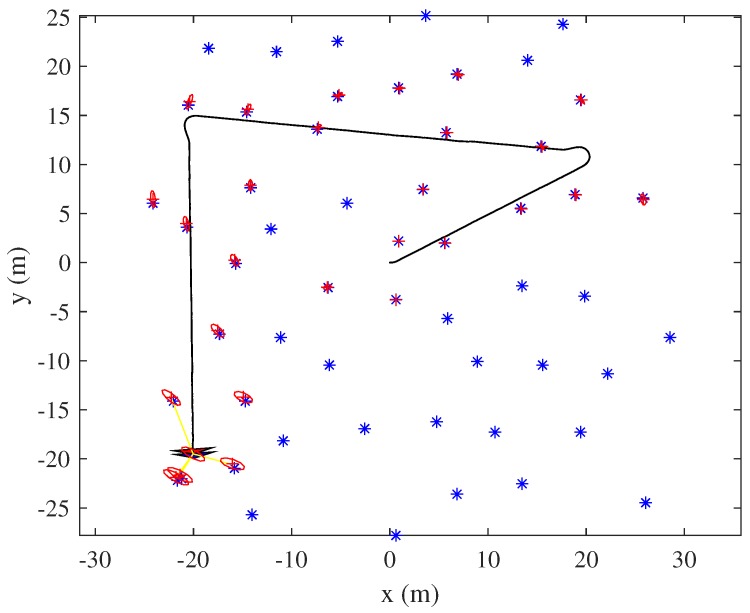
An example of extended Kalman filter (EKF)-simultaneous localization and mapping (SLAM) robot/landmark state when revisiting commences. The red ellipses reflect the covariances of registered landmarks; the red stars show the estimated positions of such landmarks; and the blue stars indicate the true positions of unregistered (unmapped) landmarks.

Figure 1 shows a sample EKF-SLAM state (of both the robot and the landmarks) when uncertainty has increased after traveling a long distance. The initial robot location was (0, 0 m). The estimated positions of landmarks that were observed early exhibit low uncertainties (represented by the volumes of the ellipses). We can find that landmark uncertainty increases as the robot travels. Revisiting is necessary to enhance the positional accuracies of both registered landmarks and the robot; the VLU grid map is obtained using the landmark state.

#### Visibility Convolution

**Figure 2:**
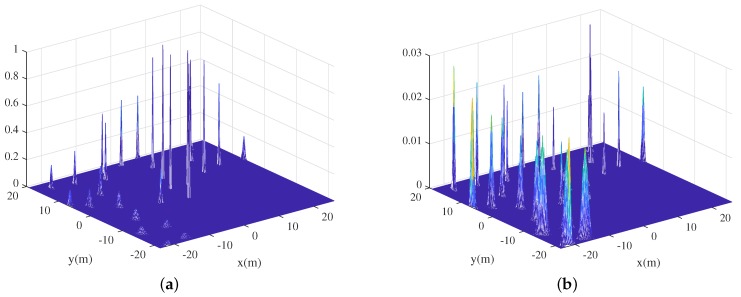
The distribution of each landmark on the gridmap: (**a**) The Gaussian distribution of each landmark. (**b**) The Gaussian distribution multiplied by the trace value of each landmark.

We now present an efficient method to calculate Equation (2) using an image convolution process. In Equation (2), the visibility of the *n*-th landmark from each grid cell is the estimated mean value of the *n*-th landmark $\left[m_{n}\left(x\right),m_{n}\left(y\right)\right]$. We improve the calculation of Equation (2) using the Gaussian distribution of each landmark and an efficient image convolution method that reflects uncertainties in the estimated landmark states. After initializing the gridmap using the size of the currently mapped area, a Gaussian distribution is inserted at each registered landmark position using both the mean value and the covariance matrix. Figure 2a shows the Gaussian distribution of each landmark depicted in Figure 1. Next, each distribution is multiplied by the trace value of its covariance matrix. Figure 2b shows the trace-multiplied Gaussian distribution map.

**Figure 3:**
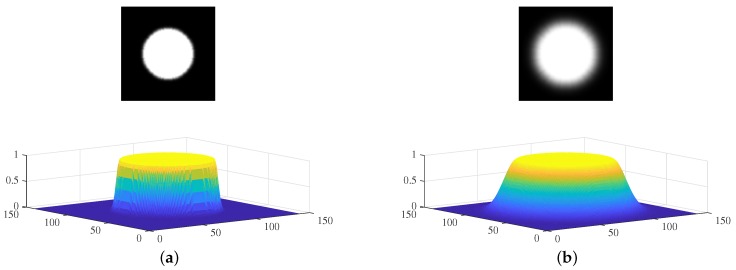
The sensor uncertainty kernels used for visibility convolution: (**a**) A convolution kernel with small sensor errors at the maximum range grid cells. (**b**) A convolution kernel with large sensor errors at the maximum range grid cells.

The two distribution gridmaps are subjected to simple image convolution. The shape of the kernel depends on the working distance, the field of view, and the sensor accuracy (the kernel is the “measurement model” of an EKF-SLAM). Figure 3 shows two kernels used to calculate convolutions. We assume that the robot features a 360${}^{\circ }$ field of view sensor; the kernel can thus be circular. If the robot features a 120${}^{\circ }$ sensor, the kernel can be fan-shaped. Within the maximum range, the sensor uncertainty kernel value is 1 because the sensor can detect the landmarks that are within the maximum range. At the maximum range, near the end of the sensor ray, the kernel value follows a Gaussian distribution that considers errors in sensor measurements. Figure 3a shows a convolution kernel with a small sensor error. Figure 3b shows a kernel reflecting a large sensor error; the deviation is large at the maximum range.

**Figure 4:**
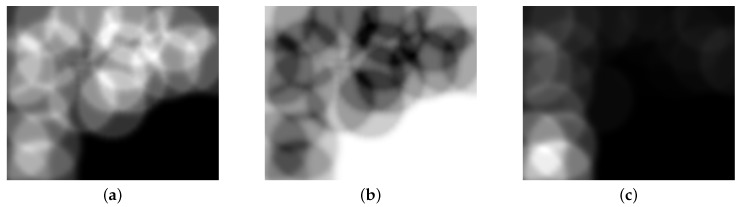
Visibility convolutions derived using sensor uncertainty kernels: (**a**) The probabilistic number of visible landmarks. (**b**) The probabilistic number of invisible landmarks. (**c**) The results of the convolution between the trace-multiplied Gaussian distribution map and the sensor uncertainty kernel.

**Figure 5:**
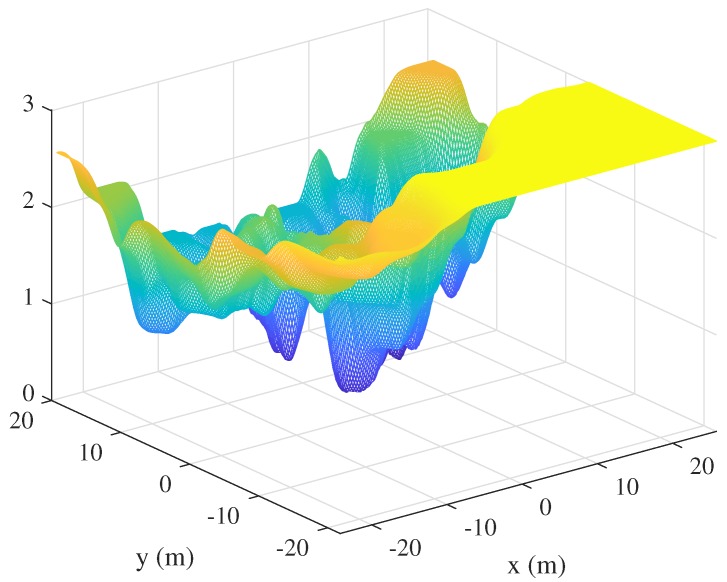
The visible landmark uncertainty (VLU) gridmap.

Using the Gaussian distribution map of the landmarks, convolution is performed with the sensor uncertainty kernel. This yields the probabilistic number of visible landmarks with consideration of the measurement uncertainty. The visibility convolution of the EKF-SLAM state of Figure 1 is shown in Figure 4. Figure 4a is the convolution between the Gaussian distribution of each landmark in Figure 2a and the sensor uncertainty kernel. Lighter grids indicate that more landmarks are observed at that position. Figure 4b shows the probabilistic numbers of invisible landmarks, obtained by subtracting the probabilistic numbers of visible landmarks from the total number of registered landmarks when revisiting commences. This value is used to calculate the first term of Equation (3) via multiplication by the maximum trace value of all landmarks (thus that of the most uncertain landmark). This assigns higher values to VLU map grid positions at which the robot observes fewer landmarks. Figure 4c shows the convolution between the trace-multiplied Gaussian distribution map of Figure 2b and the sensor uncertainty kernel; this corresponds to the second term of Equation (3). The fewer the number of landmarks detected, and the more uncertain their positions, the lighter the color and the higher the uncertainty. The final VLU map is shown in Figure 5. Overall, the areas in which less uncertain landmarks are visible have lower values and can be considered as revisiting positions to increase the accuracy of landmark positioning and robot pose by correct data association.

### 2.2. Selection of Revisiting Positions Using the VLU Map

The VLU grid map is used to select several revisiting positions. Each position must feature landmarks of low uncertainty to enhance the probability of correct data association. Optimally, the robot will detect not only accurate but also somewhat uncertain landmarks. This may appear to be contradictory. However, this reduces the overall uncertainties of state estimation and increases exploration efficiency; map information must be extended. To select positions at which the robot can achieve these aims, we select the less uncertain registered landmarks. Any selected landmark will be visible from several positions, and these positions have relatively lower VLU values than the positions where the selected landmarks are not visible; the position with the highest VLU value among these several positions is chosen for revisiting.

Algorithm 1 shows how to select revisiting positions using the VLU gridmap. The less uncertain landmarks are identified by Equation (5). A landmark for which the covariance matrix exhibits a trace value greater than the mean of the overall landmark covariance matrix is selected as a re-observation target. Next, for each selected landmark $l_{k}$, we define grid cells from which $l_{k}$ is visible (lines 2–9). When there are several grid cells where one selected landmark is visible, the grid cell with the highest VLU is selected as a revisiting position for both data association and exploratory utility. If that cell has been already selected for viewing of another landmark, the cell with the next-highest VLU is selected (11–19). Thus, Algorithm 1 runs until a grid cell with a high VLU that is not assigned to another landmark is chosen for each landmark.

**Algorithm 1** Selecting revisiting locations**Require:**
all grid cell indices $\left\{g\left(i,j\right)\right\}$, VLU gridmap $\left\{g_{VLU}\left(i,j\right)\right\}$, the covariance matrices of all *N* registered landmarks $P=\left\{P_{1},\ldots ,P_{N}\right\}$**Ensure:**
Revisiting positions Loc
1:Select the less uncertain landmarks according to the trace of its covariance matrix
(5)$$\begin{array}{c} \mathrm{Landmark}\;k,\;l_{k},\;\mathrm{with}\;trace\left(P_{k}\right)<\frac{1}{N}\sum_{i=1}^{N}trace\left(P_{i}\right) \end{array}$$
$$\begin{array}{c} \begin{array}{c} N:\mathrm{total}\mathrm{number}\mathrm{of}\mathrm{registered}\mathrm{landmarks}\\ P_{i}:\mathrm{covariance}\mathrm{matrix}\mathrm{of}\mathrm{landmark}i \end{array} \end{array}$$2:**for** all selected landmarks, $l_{k}$ **do**3:    **for** all grid cells, $g\left(i,j\right)$ **do**4:        **if** $l_{k}$ is visible at $\left\{g_{i,j}\left(x,y\right)\right\}$ **then**5:           Push $g\left(i,j\right)$→$G_{k}$6:        **end if**7:    **end for**8:    Push $G_{k}$→G9:
**end for**
10:
Loc←∅
11:**for** all elements, $G_{k}$ of G **do**12:    Select $g\left(i,j\right)$ which has the highest $g_{VLU}\left(i,j\right)$ among all elements of $G_{k}$13:    **if** there is not $g\left(i,j\right)$ in Loc **then**14:        Push $g\left(i,j\right)$→Loc15:    **else**16:        Remove $g\left(i,j\right)$ from $G_{k}$17:        **goto** Step 1118:    **end if**19:
**end for**



## 3. The Covariance Traveling Salesman Problem

In the previous section, we calculated the VLU of each map position, and used this to select revisiting positions. We employ a solution of the TSP algorithm to define the revisiting order. The TSP is an extensively explored optimization problem that deals with a salesman who requires the shortest path between *N* cities or nodes [32]. In terms of acquiring the shortest path, the algorithm can be used to optimize revisiting or to explore robotic frontiers [33]. As the odometry error increases as the robot moves, the shorter the distance traveled to re-detect each landmark, the better the SLAM state estimation.

### 3.1. Traveling Salesman Problem: A Brief Review

When a salesman must visit several cities, starting from and returning home, he needs to minimize his total travel distance. It is assumed that the salesman knows where all the cities are and the traveling costs between them. The constraints are: (i) each city may be visited only once; and, (ii) the salesman must return home (closing a loop). We view the mobile robot revisiting problem as a two-dimensional (2D) TSP where the traveling distance is Euclidean or reflects the navigation cost between revisiting positions. The classical formulation of the TSP follows. Let:

(6) $$\begin{array}{c} x_{ij}=\left\{\begin{array}{cc} 1 & \mathrm{if}\;\mathrm{the}\;\mathrm{tour}\;\mathrm{uses}\;a\;\mathrm{leg}\;\mathrm{between}\;i\;\mathrm{and}\;j\\ 0 & \mathrm{if}\;\mathrm{not} \end{array}\right.. \end{array}$$

This minimizes:

(7) $$\begin{array}{c} Z=\sum_{i}\sum_{j>i}c_{ij}x_{ij} \end{array}$$

when:

(8) $$\begin{array}{c} \sum_{j<i}x_{ji}+\sum_{j>i}x_{ij}=2,\mathrm{for}\mathrm{all}i \end{array}$$

(9) $$\begin{array}{c} \sum_{i\in S}^{}\sum_{j\notin S}x_{ij}+\sum_{i\notin S}^{}\sum_{j\in S}x_{ij}\geq 2, \end{array}$$

$$\begin{array}{c} \mathrm{for}\mathrm{all}\mathrm{proper}\mathrm{subset}\;S,|S|\geq \;3\\ x_{ij}=\mathrm{binary},\mathrm{for}\;\mathrm{all}\;i;j>i. \end{array}$$

**Figure 6:**
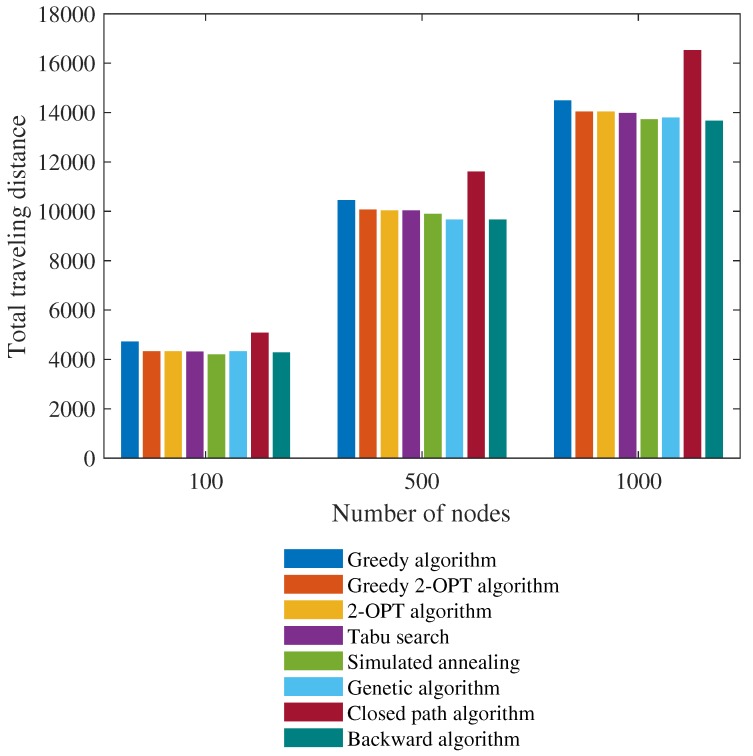
Comparison of the traveling salesman problem (TSP) solutions using various heuristic algorithms.

Equation (8) deals with constraint (i) and Equation (9) deals with the loop-closing constraint (ii). The TSP is probably the best-known optimization problem but it is unlikely that it can be efficiently solved using a polynomial time algorithm. As all $O\left(n!\right)$ potential permutations of the cities must be examined, computation is difficult when the number of cities is large. Although the TSP cannot be fully solved in polynomial time, a near-optimal solution can be acquired with the aid of heuristics [32,34]. A heuristic algorithm commences by selection of a single city, followed by addition of all remaining cities one-by-one; this is a ‘greedy algorithm’. An improved heuristic algorithm is based on the local-search concept, analyzing transitions from one solution to the next in a search for better solutions. We tested various local heuristic algorithms when seeking TSP solutions to mobile robot revisiting. Figure 6 shows the total traveling distances of eight algorithms. The average computational time of seven algorithms (excluding the backward algorithm) was 0.1 s for 100 cities, 4 s for 500 cities, and 7 s for 1000 cities. These values are acceptable when analyzing mobile robot revisiting.

### 3.2. Traveling Costs between Revisiting Positions

After choosing positions to be revisited, the traveling costs are computed. The positions form the nodes of the TSP and the traveling costs between each pair of positions form the cost matrix of the TSP, thus $c_{ij}$ of Equation (7). Traveling costs of robotic navigation can be defined in various ways, including the geometric Euclidean distance or the obstacle-free shortest path. If the robot features perfectly accurate sensors (including a perfect wheel odometer), the Euclidean distance can serve as the shortest total revisit distance. However, sensors are never perfect, and SLAM deals with sensor errors. Also, it is important to not only minimize the total travel distance but also to reduce the uncertainty of the overall SLAM state. Such uncertainty depends on both the path and new sensor measurements. We propose the covariance TSP that defines each between-position travel cost as the expected covariance change of the robot after it moves from one revisiting position to another.

SLAM algorithms, especially EKF-SLAM, engage in both prediction and update processes [22]. During the prediction process, the robot state is computed by the motion model and the control inputs. The update process can be performed when sensor observations are available. After an observation is made, the innovation (the difference between the actual and predicted observations as revealed by the observational model) and the robot state evaluated during prediction must be calculated. However, because we are now considering a kind of path planning problem before actual movement, it is impossible to obtain real observations prior to arrival. Therefore, we simulate the observations expected as the robot travels between two revisiting positions. In these simulations, the landmark state is that at the time a revisit is scheduled. The initial covariance of the robot state serves as the zero matrix.

**Figure 7:**
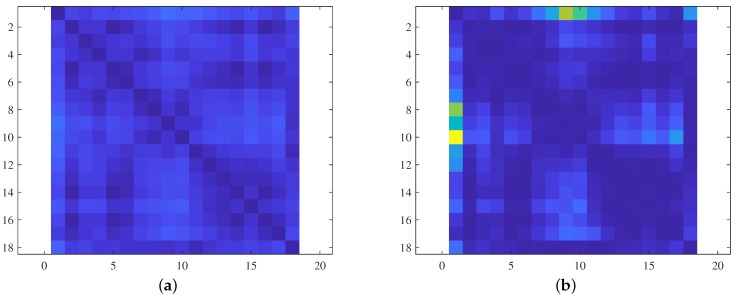
Cost matrices of TSP algorithms: (**a**) A Euclidean cost matrix. (**b**) A covariance cost matrix.

The resulting covariance cost matrix is non-symmetric because the measurements obtained during travel from nodes “A” to “B” and “B” to “A” differ. Although the observed landmarks are the same, the covariance is computed differently because the order of measurements varies. Figure 7 shows examples of cost matrices; Figure 7a is the cost matrix derived using the Euclidean distance between nodes and Figure 7b is the matrix yielded by the expected covariance change. When that expected covariance change serves as the travel cost, we can find a revisiting route that minimizes the covariance change. Therefore, the robot state uncertainty is maintained below a certain, predefined low value during revisiting.

With the covariance cost matrix, a genetic algorithm (GA) was used to solve the TSP in this paper. GA is intuitively very simple and is known to perform very well for small-sized (n<100) TSP instances [35,36]. The proposed covariance cost matrix can be used in combination with any TSP algorithm and GA can be replaced with other heuristics and metaheuristics. The details and benchmark results of leading TSP heuristics such as the Lin–Kernighan (LK) method and the stem-and-cycle (S&C) method can be found in [37]. Metaheuristics have been also widely used to solve TSP and the vehicle routing problem (VRP) [38,39].

## 4. Simulation Results

We simulated our revisiting method to verify performance in terms of SLAM covariance reduction. We evaluated the robot pose and the positions of registered landmarks. We did not consider the problem of obstacles in the environment. This is the problem of local planners and there are various algorithms to deal with this. The robot’s laser range finder had a scanning range of 360${}^{\circ }$ and a maximum range of 7 m. Robot locomotion featured a differential drive constraining movement within each time step. Using range data and wheel odometry, the EKF-SLAM algorithm estimates the state of the robot and landmarks. The environment is completely unknown before the SLAM algorithm commences.

The initial robot position was (0, 0 m) and the robot moved to (20, 10 m), (−20, 15 m), and (−20, −20 m). On arrival at the third position, the SLAM covariance became large. To reduce uncertainty, the robot revisited the initial position using backtracking or planned the revisiting path by the proposed method. During backtracking, the revisited positions were placed every 2 m along the past trajectory. Using our method, the positions to be revisited were chosen by reference to the VLU map and the covariances of registered landmarks; the revisiting sequence was calculated with the aid of the proposed covariance TSP algorithm with GA.

**Figure 8:**
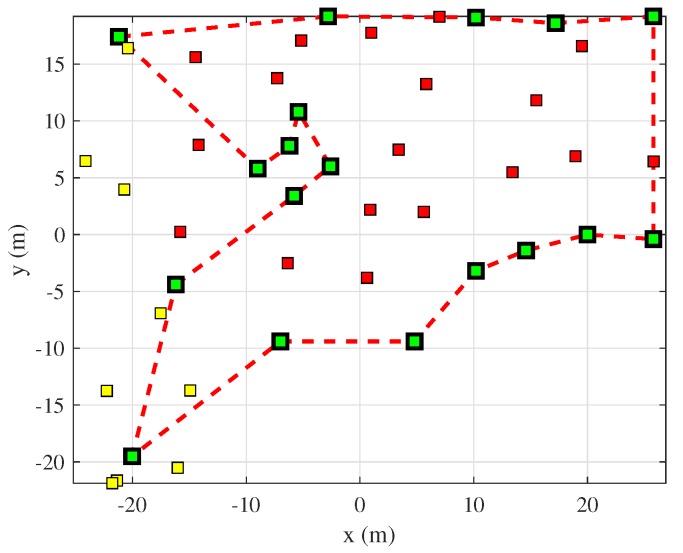
A revisiting path constructed using the proposed covariance TSP algorithm. The yellow squares are non-selected landmarks; the red squares are landmarks selected for revisiting; the green squares are the revisiting positions dictated by the VLU grid map; the red dotted line is the optimal revisiting path defined by the covariance TSP algorithm.

Figure 8 shows the landmarks selected by, and the revisiting path proposed by, our method. The red and yellow squares are registered landmarks of the EKF-SLAM state at the time revisiting was planned. The yellow squares are non-selected landmarks. The red squares are landmarks selected by Equation (5). These are “less uncertain” landmarks that must be re-observed to reduce the overall SLAM uncertainty. The green squares are the revisiting positions at which the robot can detect the selected landmarks. These positions have low VLU values; non-mapped areas can be scanned to expand map information.

**Figure 9:**
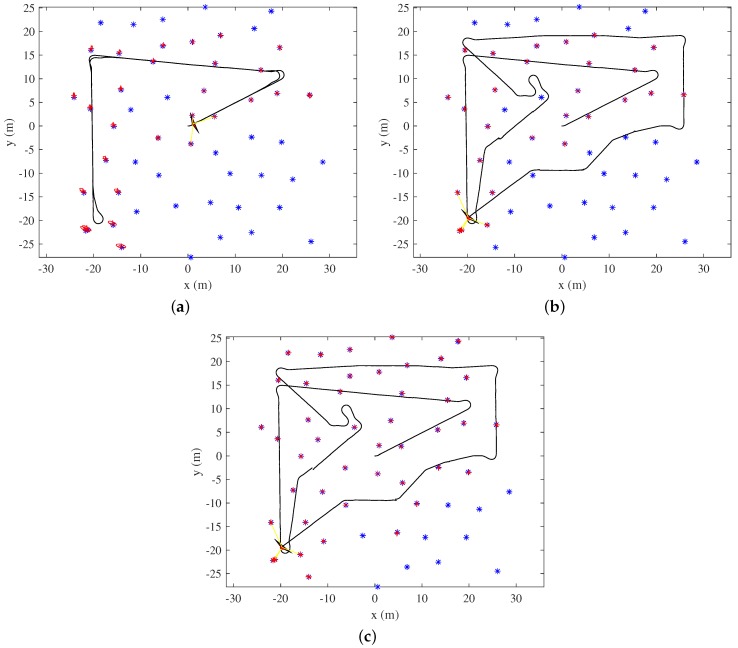
The simulation results of three revisiting methods: (**a**) Backtracking. (**b**) The covariance TSP${}_{\mathrm{fixed}}$ algorithm that restricts adding new landmarks during revisiting. (**c**) The covariance TSP algorithm.

Figure 9 shows the final EKF-SLAM state after revisiting either via a backtracking algorithm (Figure 9a) or with the aid of the proposed covariance TSP algorithm. For revisiting by the covariance TSP, two approaches were applied. In the first covariance TSP algorithm, which is denoted by Covariance TSP${}_{\mathrm{fixed}}$, adding new landmarks to the SLAM state was not permitted during the revisiting process (Figure 9b). The number of registered landmarks was kept the same from the beginning of revisiting to the end. Therefore, the number of landmarks during Covariance TSP${}_{\mathrm{fixed}}$ revisiting was almost the same as the number of landmarks during backtracking. In the second covariance TSP algorithm, Covariance TSP, new landmarks were registered to the SLAM state when the robot obtained new observations (Figure 9c) during revisiting. The volume of the ellipse at each landmark reflects the uncertainty. As the robot obtained almost the same measurements for each registered landmark during backtracking, the uncertainties of landmarks near the position at which revisiting commenced are larger than those of early-registered landmarks. Using the proposed method, the robot moved along a closed TSP path and obtained different measurements for the re-observed landmarks and the newly detected landmarks that were able to make the correct data associations for the SLAM algorithm. Consequently, the overall uncertainty of the landmark was effectively reduced.

**Figure 10:**
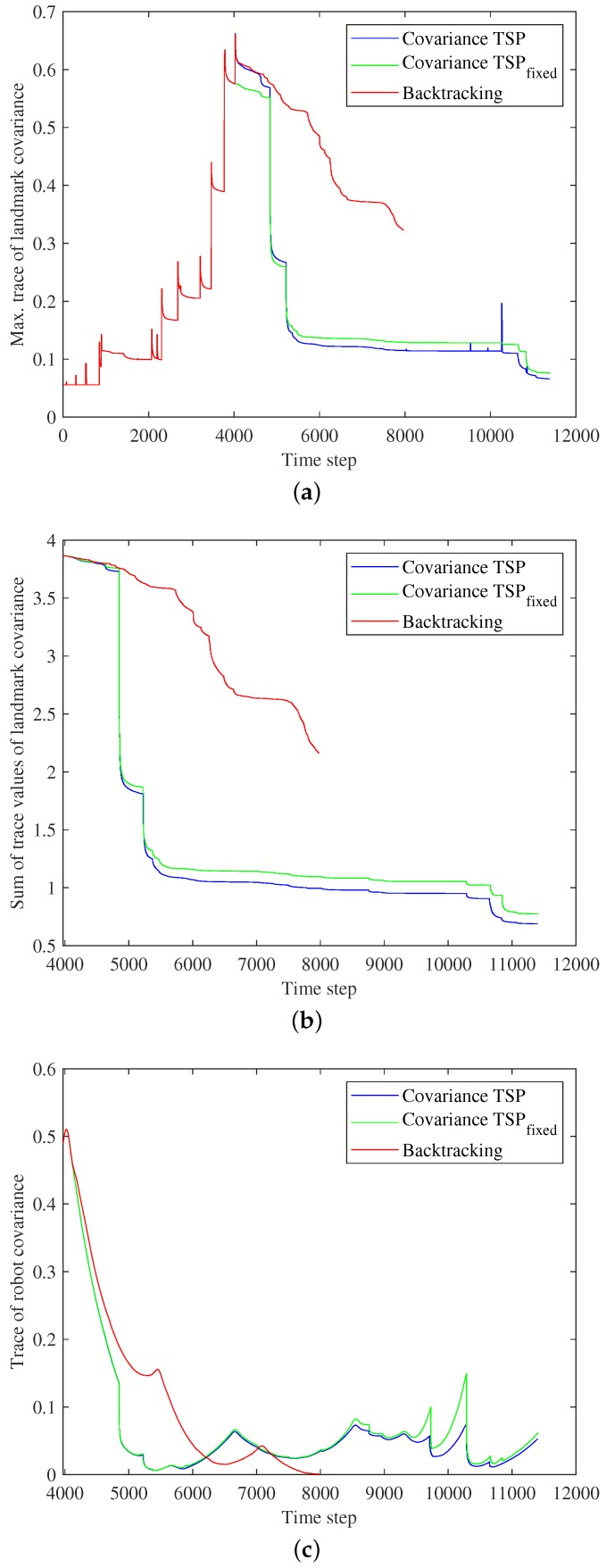
Comparisons of the proposed methods and backtracking: (**a**) The maximum trace value of landmark covariance matrix. (**b**) The sum of the trace values of the landmarks registered until the time when revisiting was planned. (**c**) The trace of the robot covariance matrix.

**Figure 11:**
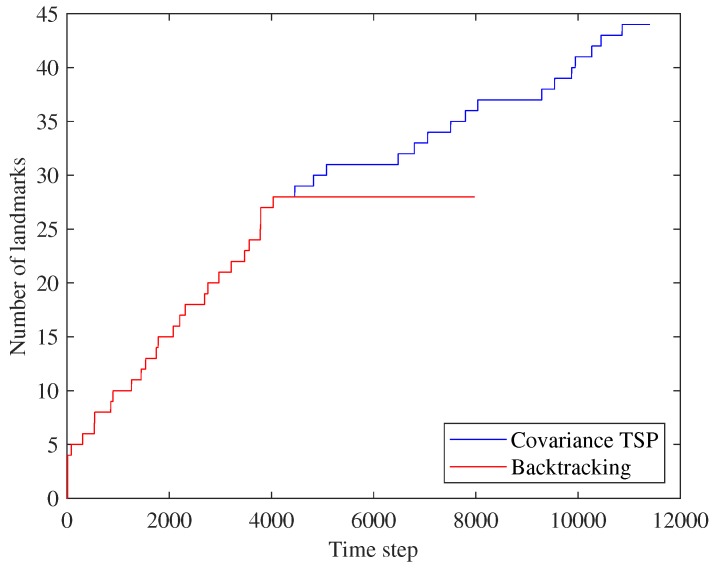
The number of registered landmarks.

Figure 10 and Figure 11 compare the results of backtracking and use of the proposed covariance TSP algorithms. Revisiting commenced at step 3972. Figure 10a shows the maximum trace value among all landmarks at each time step, and we can compare the uncertainty of the most uncertain landmark identified by backtracking and the covariance TSP algorithms from SLAM commencement to completion of revisiting. As the landmark state was identical for three algorithms until revisiting commenced, the maximum trace values are also the same. After revisiting began, we explored how effectively the three methods reduced landmark uncertainty. As the overall distance traveled during backtracking was shorter than that of covariance TSP revisiting, the robot stopped the algorithm at step 7975 of backtracking and step 11,402 of Covariance TSP and Covariance TSP${}_{\mathrm{fixed}}$. The ultimate, maximum trace values of Covariance TSP and Covariance TSP${}_{\mathrm{fixed}}$ are much smaller than that of backtracking; our algorithm thus more effectively reduced uncertainty. Moreover, even at the end of backtracking (step 7975), the maximum trace values of our algorithms were smaller because they fell dramatically over time. At about step 10,000, the trace of Covariance TSP rose but then immediately decreased. This was because the robot obtained not only measurements of the landmarks registered when planning the revisiting path but also measurements of new landmarks that were relatively uncertain in terms of SLAM registration. These new landmarks became less uncertain via the EKF-SLAM updating process. As loop-closing by re-observing the already registered landmarks occurred and any new landmarks were not added in Covariance TSP${}_{\mathrm{fixed}}$ revisiting, the maximum trace consistently decreased until completion of revisiting. However, because the number of landmarks increased and it affected the overall uncertainty in Covariance TSP, the maximum trace was smaller than that of Covariance TSP${}_{\mathrm{fixed}}$.

In Figure 10b, we also compared the sum of the trace values of the landmarks registered to the SLAM state at the time when revisiting was planned, step 3972. The proposed algorithms outperformed backtracking in terms of reducing and maintaining landmark uncertainty. Although the proposed revisiting path was longer than that of backtracking, our methods reduced the overall landmark uncertainty earlier than backtracking. Therefore, it is possible to cease revisiting in the middle of the planned path if the overall uncertainty falls to the desired value.

Figure 10c shows the covariance trace of the robot during revisiting. In the result of backtracking, when the robot was at the initial position (0, 0 m), the robot covariance became almost the zero matrix, that is the initial covariance matrix. With our methods, the robot pose uncertainty decreased more rapidly than during backtracking at early revisiting steps. Although the robot pose uncertainty of the proposed revisiting algorithms was larger than that of backtracking, the value was maintained at relatively low values compared to that when revisiting commenced. This was because the revisiting path of the covariance TSP solution does not contain the initial position (0, 0 m) as a node; the nodes are the positions from which less uncertain landmarks are highly observable.

Figure 11 shows the overall numbers of landmarks in the SLAM state. The proposed method, Covariance TSP, selects revisiting positions that ensure both data association (by re-observing registered landmarks) and exploratory utility (by adding new landmarks). The number of landmarks does not change during backtracking. The number of landmarks rises as new positions are visited and new measurements obtained along the TSP path of the proposed method.

**Figure 12:**
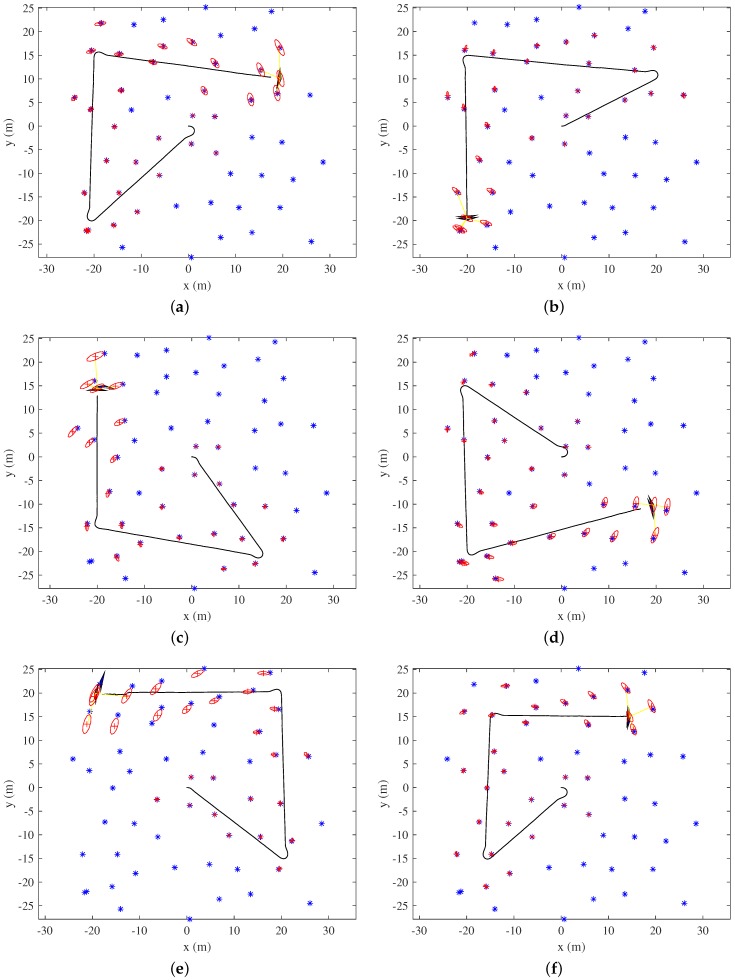
Examples of EKF-SLAM robot/landmark state when revisiting commenced: (**a**–**f**) The EKF-SLAM state used for simulations. After traveling through three positions, uncertainty has increased. Ten simulations for each SLAM state were performed using Covariance TSP${}_{\mathrm{fixed}}$ and Covariance TSP, respectively.

For further validation of the proposed method, multiple simulations using different SLAM states in the same environment were performed. Figure 12 shows six SLAM states when revisiting commenced. After traveling through three positions, the uncertainty of the robot pose and the landmark positions have increased. It is worth noting that revisiting paths can be different even with the same SLAM state since GA incorporates randomness. Ten simulations for each SLAM state were performed by using Covariance TSP${}_{\mathrm{fixed}}$ and Covariance TSP, respectively; total 120 simulations were performed by using the proposed algorithms. The average, minimum, and maximum values for each ten simulations were calculated. Because the backtracking path was determined along the previous trajectory, one simulation by backtracking was performed for each SLAM state.

**Figure 13:**
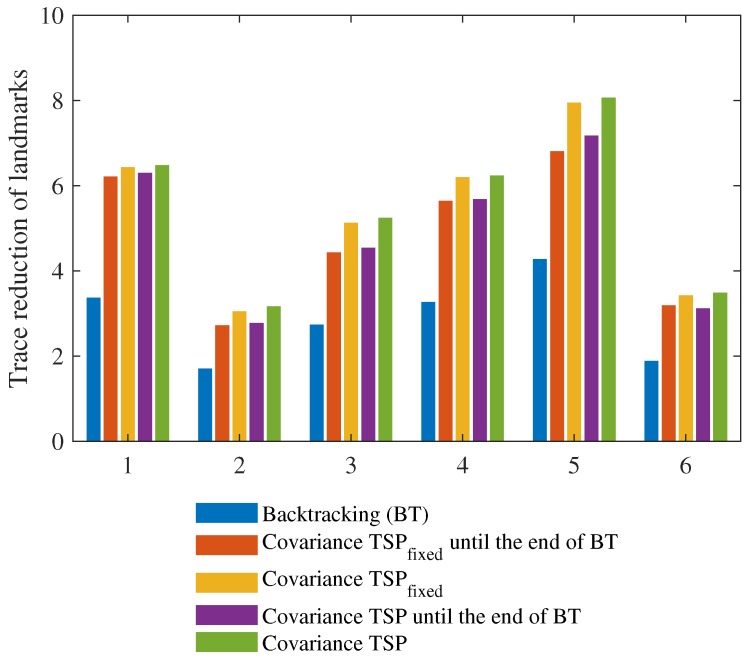
The reduction of the trace sum of the landmarks by backtracking and the average reduction of the trace sum of the landmarks by Covariance TSP${}_{\mathrm{fixed}}$ and Covariance TSP.

Figure 13, and Table 1 and Table 2 show the reduction of the trace sum of the landmarks registered in the SLAM state at the time when revisiting was planned and started. The sum of trace values of the landmarks for a single simulation is shown in Figure 10b, and the reduction was calculated as follows:

(10) $$\begin{array}{c} \sum_{n=1}^{N}trace\left(P_{n}^{Start}\right)-\sum_{n=1}^{N}trace\left(P_{n}^{End}\right) \end{array}$$

where *N* is the number of the landmarks in the SLAM state at the time when revisiting was planned, $P_{n}^{Start}$ is the covariance matrix of the *n*-th landmark at the time of starting revisiting, and $P_{n}^{End}$ is the covariance matrix of the *n*-th landmark at the end of revisiting. The reduction of the trace sum means reduced uncertainty by each revisiting algorithm. Table 3 and Table 4 show the ratio of the trace sum at the start of revisiting to the end of revisiting, and it was calculated as follows:

(11) $$\begin{array}{c} \frac{\sum_{n=1}^{N}trace\left(P_{n}^{End}\right)}{\sum_{n=1}^{N}trace\left(P_{n}^{Start}\right)}. \end{array}$$

The ratio of the trace sum means how efficiently each revisiting algorithm reduces overall uncertainty in the registered landmarks. As more uncertainty has been reduced during the revisiting process, the ratio is smaller. The results of Table 1 and Table 3 are obtained by the simulations from the beginning of revisiting to the end of revisiting by the proposed algorithms. The numbers in parentheses are the results from the beginning of revisiting to the time step when backtracking finished.

The average reduction of the trace sum by Covariance${}_{\mathrm{fixed}}$ TSP and Covariance TSP in Table 1 for all 120 simulations is larger than that by backtracking in Table 2. The average ratio of the final trace sum to the starting trace sum by the proposed methods in Table 3 is smaller than that by backtracking in Table 4. A few results in parentheses are larger than the backtracking results. However, these values have been reduced when revisiting is completed.

## 5. Conclusions

We developed a revisiting method reducing the overall uncertainty of the SLAM state. Existing revisiting approaches simply select revisiting positions from previous trajectories or return to the initial positions, whereas the proposed method selects revisiting positions with consideration of both landmark and sensor uncertainty. To do this, we proposed VLU grid maps showing the landmark visibility from each grid cell position. Less-uncertain landmarks were then defined, and revisiting positions from which the robot could not only re-observe selected landmarks but also detect new landmarks (to expand map information) were selected. Travel among revisiting positions was optimized using the TSP algorithm. As our covariance TSP algorithm employs covariance changes between pairs of nodes, the revisiting path efficiently reduces the overall covariance, not the total distance traveled.

We simulated the proposed method and simple backtracking. The proposed method reliably reduced the overall uncertainty of the EKF-SLAM state (the robot pose and the landmark positions). Although the closed path of the proposed method is longer than that of backtracking, our method reduces overall uncertainty earlier, and more extensively, than does backtracking. This is because the proposed revisiting path directs the robot to a place that allows valuable data association. Therefore, the proposed covariance TSP revisiting method can efficiently reduce and control the overall state uncertainty of landmark-based SLAM problems.

## Figures and Tables

**Table 1:** The reduction of the trace sum of the landmarks registered in the SLAM state at the time when revisiting was planned and started.

		1	2	3	4	5	6
Covariance TSP (fixed)	Min.	6.40 (5.49)	2.96 (2.47)	5.04 (2.00)	6.14 (4.34)	7.82 (3.61)	3.40 (3.11)
	Avg.	6.43 (6.21)	3.05 (2.72)	5.13 (4.43)	6.20 (5.64)	7.95 (6.81)	3.43 (3.19)
	Max.	6.48 (6.41)	3.12 (2.80)	5.24 (4.98)	6.27 (6.08)	8.00 (7.23)	3.46 (3.25)
Covariance TSP	Min.	6.46 (5.75)	3.15 (2.61)	5.17 (2.33)	6.22 (4.19)	7.96 (4.89)	3.47 (1.33)
	Avg.	6.48 (6.30)	3.17 (2.78)	5.25 (4.54)	6.24 (5.68)	8.07 (7.17)	3.49 (3.12)
	Max.	6.50 (6.40)	3.19 (2.87)	5.35 (5.27)	6.28 (6.02)	8.18 (7.94)	3.57 (3.36)

**Table 2:** The reduction of the trace sum of the landmarks registered in the SLAM state at the time when revisiting was planned and started.

	1	2	3	4	5	6
Backtracking	3.37	1.71	2.74	3.27	4.27	1.89

**Table 3:** The ratio of the trace sum at the start of revisiting to the end.

		1	2	3	4	5	6
Covariance TSP (fixed)	Min.	0.09 (0.10)	0.19 (0.28)	0.11 (0.15)	0.10 (0.13)	0.09 (0.18)	0.14 (0.20)
	Avg.	0.09 (0.12)	0.21 (0.30)	0.13 (0.25)	0.11 (0.19)	0.10 (0.23)	0.15 (0.21)
	Max.	0.10 (0.23)	0.24 (0.36)	0.14 (0.66)	0.12 (0.38)	0.11 (0.59)	0.16 (0.23)
Covariance TSP	Min.	0.08 (0.10)	0.18 (0.26)	0.09 (0.10)	0.10 (0.13)	0.07 (0.10)	0.12 (0.17)
	Avg.	0.09 (0.11)	0.18 (0.28)	0.11 (0.23)	0.10 (0.18)	0.09 (0.19)	0.14 (0.23)
	Max.	0.09 (0.19)	0.19 (0.33)	0.12 (0.60)	0.11 (0.40)	0.10 (0.45)	0.14 (0.67)

**Table 4:** The ratio of the trace sum at the start of revisiting to the end.

	1	2	3	4	5	6
Backtracking	0.52	0.56	0.53	0.53	0.52	0.53
